# Human Milk Feeding in Preterm Infants: What Has Been Done and What Is to Be Done

**DOI:** 10.3390/nu12010044

**Published:** 2019-12-23

**Authors:** Elvira Verduci, Maria Lorella Giannì, Alessia Di Benedetto

**Affiliations:** 1 Department of Pediatrics, Hospital San Paolo, University of Milan, 20142 Milan, Italy; alessia.dibenedetto@unimi.it; 2 Fondazione IRCCS Ca’ Granda Ospedale Maggiore Policlinico, NICU, 20142 Milan, Italy; maria.gianni@unimi.it; 3 Department of Clinical Science and Community Health, University of Milan, 20142 Milan, Italy

**Keywords:** breastfeeding, human milk, human donor milk, promotion of breastfeeding, preterm infant, VLBW

Human milk is recommended as the natural feeding for both term and preterm infants for the several health benefits associated with its consumption. The importance of human milk is not only limited to its nutritional value but it also includes the provision of bioactive factors involved in the optimization of infants’ growth and neurodevelopment. A huge amount of researches indicates that the biological effects of human milk are dose dependent. Moreover, an increasing number of studies are showing that breast milk contains stem cells whose implications in infant neurodevelopment are under study. Hence, it is mandatory to promote initiation of breastfeeding as well as to support its duration [1,2,3,4].

When taking into consideration feeding options for preterm infants, it should be highlighted that there is a biological hierarchy with milk of own mothers’ being the first choice. When own mothers’ milk is not available, donor human milk is recommended, whereas formula feeding should be the last choice [2].

Human milk feeding represents a cost-effective strategy to reduce disease impact and associated costs in preterm infants, during the neonatal intensive care unit hospitalization [5]. Colaizy estimated that suboptimal feeding of extremely low birth weight infants would cost $27.1 million (CI $24 million, $30.4 million) in direct medical costs, $563,655 (CI $476,191, $599,069) in indirect nonmedical costs, and $1.5 billion (CI $1.3 billion, $1.6 billion) in cost attributable to premature death [6].

The systematic review and meta-analysis published by Miller [7] summarized the available evidence in relation to the dose-dependent effect of human milk feeding on decreasing morbidity in infant born <28 weeks’ gestation and/or birth weight <1500 g. Recent studies have assessed the different impact of human milk and preterm infant formula (PTF) feeding on one single outcome, thus limiting the in-depth analysis of the synergic protective co-effects led by the different nutritional and non-nutritional components of human milk against the most frequent comorbidities that affect premature infants. Remarkably, Miller considered the combination of five major morbidities (necrotizing enterocolitis, late onset sepsis, bronchopulmonary dysplasia, retinopathy of prematurity and neurodevelopment), pointing out the clear protective effect of human milk against necrotizing enterocolitis and possible reduction in late onset sepsis, severe retinopathy of prematurity and severe necrotizing enterocolitis. The comparison between various combinations of mixed feeding, human milk and preterm infant formula allowed for a specific investigation on the dose effect associated with human milk feeding.

However, no conclusive evidence has been drawn on the incidence of bronchopulmonary dysplasia and on neurodevelopment, thus underlining the need for implementing research towards this direction.

This systematic review allows for gaining further insights on the importance of human milk to improve the outcomes in preterm neonates. Within this context, it must be remembered that benefits of human milk include improvement of feeding tolerance, risk-reduction of re-hospitalization due to infectious diseases, and neurodevelopment optimization. Thus, there is wide consensus in recognizing human milk feeding as a cost-effective strategy in order to reduce economic costs related to prematurity and to aim to a personalized therapy improving the quality of care in these infants [8]. These characteristics call for considering human milk promotion and support in these vulnerable infants as a public health issue.

Human donor milk has been advocated as the first alternative in preterm infants when own mother’s milk is not available. Even though it has been criticized for potentially being not cost effective, human donor milk should be considered and implemented as a “bridge” to own mothers’ milk, in consideration of the individual-specificity of human milk and of the importance of breastfeeding for the mother’s health [9].

In conclusion, health care professionals should be educated on the important role of human milk in reducing the risk for developing comorbidities in preterm infants and should promote and protect breastfeeding, supporting the mothers particularly in the first 14 days after delivery. Indeed, during this window of time, the preterms’ mothers are at higher risk for producing an insufficient volume of milk, thus experiencing the so called “impaired coming to volume”, which negatively affects the long term lactation outcomes [10] in the broadest context possible. Future research on how tp implement the starting and the duration of human milk feeding in preterm infants is needed.
